# Seroprevalence of *Capripoxvirus* infection in sheep and goats among different agro-climatic zones of Odisha, India

**DOI:** 10.14202/vetworld.2018.66-70

**Published:** 2018-01-26

**Authors:** Abhishek Hota, Sangram Biswal, Niranjana Sahoo, Gnanavel Venkatesan, Sargam Arya, Amit Kumar, Muthannan Andavar Ramakrishnan, Awadh Bihari Pandey, Manoranjan Rout

**Affiliations:** 1 Department of Veterinary Epidemiology & Preventive Medicine, College of Veterinary Science and Animal Husbandry, O.U.A.T., Bhubaneswar - 751 003, Odisha, India; 2 Division of Virology, ICAR-Indian Veterinary Research Institute, Mukteswar, Nainital, Uttarakhand - 263 138; 3 ICAR-Directorate of Foot and Mouth Disease, Mukteswar - 263 138, Nainital, Uttarakhand, India

**Keywords:** capripox, goat pox, indirect ELISA, Odisha, seroprevalence, sheep pox

## Abstract

**Aim:**

The study was undertaken to assess the prevalence of antibodies to Capripoxviruses among small ruminants of Odisha, India.

**Materials and Methods:**

A total of 500 random serum samples collected from 214 sheep and 286 goats across 10 agro-climatic zones of Odisha, were screened using whole virus antigen-based indirect ELISA for antibodies against Capripoxviruses. Results were analyzed by suitable statistical methods.

**Results:**

Screening of 500 serum samples showed seropositivity of 8.88% and 31.47% in sheep and goats, respectively, for Capripoxviruses. The prevalence rate according to agro-climatic zone ranged from 0% (North Eastern coastal plain zone) to 48.57% (North central plateau zone) for goat pox, and 0% (Western undulating zone and North central plateau) to 22.22% (South Eastern ghat zone) for sheep pox. The difference in prevalence rates among the various agro-climatic zones was statistically significant (p<0.05) for goats, but not for sheep. Antibody prevalence rates among various districts were recorded to be the highest in Jagatsinghpur (30%) for sheep pox and Dhenkanal (80%) for goat pox.

**Conclusions:**

The study revealed serological evidence of Capripoxvirus infection in sheep and goat populations in the study area, in the absence of vaccination. Systematic investigation, monitoring, and reporting of outbreaks are necessary to devise control strategies.

## Introduction

Infections of *Capripoxviruses* among small ruminants, namely, sheep pox and goat pox are OIE notifiable, acute febrile and highly contagious transboundary viral diseases [1]. Sheep pox virus (SPPV) and goat pox virus (GTPV) belong to the genus *Capripoxvirus* under subfamily Chordopoxvirinae in the family Poxviridae, along with lumpy skin disease virus, which is closely related to GTPV and SPPV [2]. Both SPPV and GTPV show host preference to their homologous hosts; however, some isolates have been shown to affect both the hosts [3]. The diseases are characterized by fever, coughing, salivation, arched back, oculonasal discharge, and edema of the eyelids followed by the progres­sive development of skin lesions all over the body. The skin lesions present as erythematous macules, vesicles, and papules which ultimately develop into scabs. Lesions may also develop on mucous membranes and on internal organs, causing systemic signs such as coughing, diarrhea, depression, emaciation, abortion, and sometimes death [4]. Significant economic losses occur in terms of reduced milk yield, decreased weight gain, abortion, poor quality wool and hides, increased susceptibility to pneumonia, fly strike, and mortalities in endemic areas [4,5].

Outbreaks of these diseases in Northern and Central Africa, Middle East and most of the Asian continent have become a major concern due to the huge economic losses caused [6]. In India, goat pox outbreak was first reported in the year 1936, and sheep pox was first reported in Bombay (1931-1932) and Mysore [7]. Since then, frequent outbreaks have been reported from several states in the country causing significant economic losses [1,8-10]. As reported in the annual report (NIVEDI, 2014-2015), a total of 3245 pox outbreaks occurred among sheep and goats during the year 2005-2013 in different states of India. The average morbidity and mortality rates have been recorded as 63.5% and 49.5%, respectively, in the country [9].

As far as the North Eastern region of India is concerned, there have not been any confirmed reports of capripox outbreaks. Outbreaks and their associated risk factors including their impact on small ruminant husbandry practices have not been well documented in Odisha state. A better understanding of the current epidemiological situation of *Capripoxvirus* infections in the state would allow the establishment of improved disease control program that would benefit small ruminant livestock owners.

The present study was undertaken to determine the seroprevalence of sheep pox and goat pox in Odisha state, bordering Andhra Pradesh, Telangana, Chhattisgarh, West Bengal, and Jharkhand. To the best of our knowledge, this appears to be the first report on seroprevalence of sheep pox and goat pox in Odisha state, India.

## Materials and Methods

### Ethical approval

As per the committee for the purpose of control and supervision of experiments on animals guidelines, studies involving the collection of field clinical samples do not require any approval from the Institute’s Animal Ethics Committee.

### Study areas and sample collection

To determine the seroprevalence of *Capripoxvirus* infections in Odisha, a random sampling was carried out between October 2015 and April 2016. A total of 500 serum samples from 214 sheep (from 25 districts) and 286 goats (from 29 districts) were collected across 10 agro-climatic zones of the state (Table-1). The farmers/veterinarians were also interviewed to obtain the history of exposure to Capripoxvirus infections and their incidences, etc. Serum samples were collected as per the standard procedures and guidelines and stored at −20°C at the Department of Veterinary Epidemiology and Preventive Medicine, C.V.Sc. and A.H., O.U.A.T., Bhubaneswar, and then sent to the Division of Virology, IVRI, Mukteswar, for testing and analysis.

**Table 1:** Seroprevalence of *Capripoxvirus* infection in sheep and goats (overall and based on sex).

Species Serum Samples	Sheep			Goat		
	
	Male	Female	Total	Male	Female	Total
Total serum taken	92	122	214	135	161	286
Positive (%)	11 (11.95)	8 (6.56)	19 (8.88)	44 (32.59)	46 (28.57)	90 (31.47)
χ^2^	1.89			0.847		
p value	0.1692			0.3574		

### Indirect ELISA for detection of antibodies against Capripoxvirus

Indirect ELISA for the detection of antibodies against goat pox and SPPV was performed as described earlier [11,12] with slight modifications. Briefly, *Capripoxvirus* antigen purified by 36% sucrose cushion ultracentrifugation was used as coating antigen in carbonate-bicarbonate buffer (Sigma, USA) at 1:100 dilution in place of 1:50 in modified version for ELISA in blocking buffer containing 5% skimmed milk powder (HiMedia, India) in washing buffer (phosphate-buffered saline containing 0.05% Tween-20). Serum samples were added in duplicates at 1:50 dilution in place of 1:100 in the modified version using blocking buffer. The plates were incubated on a shaker for 1 h at 37°C. Thereafter, the plates were washed 3 times with washing buffer. Anti-goat and anti-sheep HRPO conjugates (Sigma, USA) were used at 1:12000 and 1:4000 dilutions, respectively. The plates were incubated again on a shaker for 1 h at 37°C and then washed 3 times with washing buffer. Chromogen/substrate at 50 μl was added followed by incubation at room temperature for 8-10 min. Reactions were stopped by 1M H_2_SO_4_ and results were captured with an ELISA reader at 492 nm (Tecan Austria GmbH, Austria). The cutoff value set for goat pox was found to be 0.12 (twice of the value of known negative serum+1X SD). The OD values (mean±1X SD) of the blank control reaction and conjugate control were found to be 0.097±0.002 and 0.010±0.002, respectively [12]. The cutoff value for sheep pox was found to be 0.130 (Twice the OD value of known negative serum+1X SD). The OD values (mean±1X SD) of blank control reaction and conjugate control were found to be 0.099±0.02 and 0.009±0.002, respectively [13].

### Statistical analysis

The results were considered statistically significant at p<0.05. Chi-square test using Real Statistics Data Analysis ToolPak on Excel was used to determine the difference in susceptibility of animals with respect to sex and agro-climatic zones.

## Results

Out of 214 serum samples from sheep tested by i-ELISA, 8.88% were found to be positive with 11.95% and 6.56% in male and female sheep, respectively. Of 286 serum samples from goats, the overall prevalence was found to be 31.47% with 32.59% and 28.57% positivity in male and female serum samples, respectively(Table-1). Serological prevalence based on sex in both sheep and goats was found to be statistically insignificant (p≥0.05).

**Figure-1:**
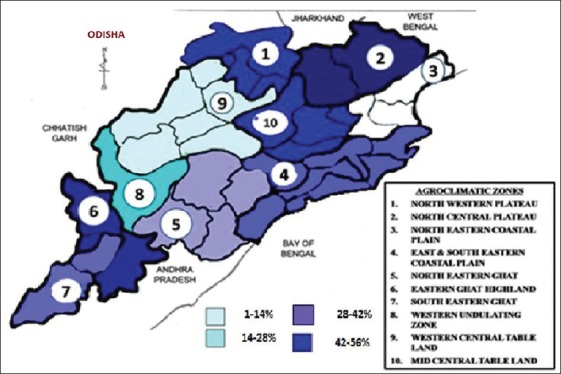
Variation in seroprevalence of goat pox among different agro-climatic zones.

**Figure-2:**
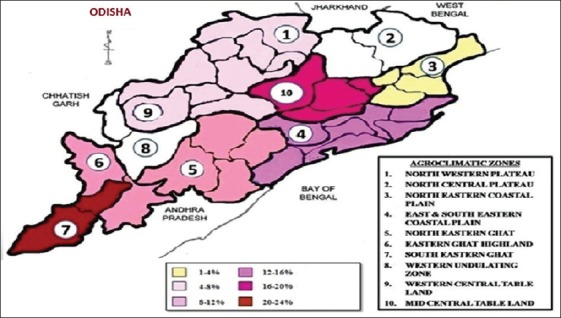
Variation in seroprevalence of sheep pox among different agro-climatic zones.

Odisha has a long border being shared with West Bengal and Chhattisgarh states with 10 agro-climatic zones. Among various agro-climatic zones, seroprevalence in goats varied from 0% (in North eastern coastal plain zone) to 48.57% (in North central plateau zone) (Figure-1), whereas in sheep it varied from 0% (in Western undulating zone and North central plateau) to 22.22% (in South eastern ghat zone) (Table-2; Figure-2). The differences in prevalence rates among the various agro-climatic zones were found to be statistically at a significant level (p<0.05) among goats, but not in sheep. Among various districts, Jagatsinghpur (30%) was recorded with the highest prevalence among sheep, whereas samples from 12 districts (Deogarh, Mayurbhanj, Balasore, Jajpur, Puri, Ganjam, Koraput, Kalahandi, Nuapada, Sonepur, Boudh, and Sambalpur) were found to be seronegative. Similarly, Dhenkanal (80%) was recorded with the highest seroprevalence among goats, while samples from 5 districts (Balasore, Bhadrak, Bolangir, Boudh, and Sambalpur) were found to be seronegative (Table-2).

**Table 2:** Seroprevalence of *Capripoxvirus* infection in sheep and goats based on agro-climatic zones and districts.

Agro-climatic zone	Climate	Districts	Positive sample/total sample taken (%)			

			Sheep	Goat	Sheep	Goat
North Western plateau	Hot and moist subhumid	Sundergarh	1/7 (14.29)	3/10 (30)	1/16 (6.25)	9/19 (47.36)
		Deogarh	0/9 (0)	6/9 (66.67)		
North central plateau		Mayurbhanj	0/15 (0)	8/15 (53.33)	0/15 (0)	17/35 (48.57)
		Keonjhar	-	9/20 (45)		
North Eastern ghat		Ganjam	0/3 (0)	1/7 (14.29)	1/11 (9.09)	12/38 (31.57)
		Gajapati	-	3/10 (30)		
		Rayagada	1/8 (12.5)	6/11 (54.54)		
		Kandhamal	-	2/10 (20)		
Western undulating zone		Kalahandi	0/9 (0)	2/11 (18.18)	0/17 (0)	5/23 (21.74)
		Nuapada	0/8 (0)	3/12 (25)		
Mid central table land		Dhenkanal	2/8 (25)	8/10 (80)	3/16 (18.75)	9/19 (47.37)
		Angul	1/8 (12.5)	1/9 (11.11)		
Western central table land		Bolangir	-	0/10 (0)	1/19 (5.26)	6/46 (13.04)
		Sonepur	0/3 (0)	1/5 (20)		
		Boudh	0/3 (0)	0/8 (0)		
		Sambalpur	0/5 (0)	0/4 (0)		
		Bargarh	-	4/11 (36.36)		
		Jharsuguda	1/8 (12.5)	1/8 (12.5)		
East and South Eastern coastal plain	Hot and Humid	Cuttack	1/9 (11.11)	6/10 (60)	8/54 (14.81)	19/50 (38)
		Jagatsinghpur	3/10 (30)	3/7 (42.86)		
		Kendrapara	1/9 (11.11)	2/7 (28.57)		
		Puri	0/11 (0)	1/10 (10)		
		Khurda	2/7 (28.57)	4/7 (57.14)		
		Nayagarh	1/8 (12.5)	3/9 (33.33)		
North Eastern coastal plain	Moist subhumid	Balasore	0/14 (0)	0/16 (0)	1/39 (2.56)	0/27 (0)
		Bhadrak	1/10 (10)	0/11 (0)		
		Jajpur	0/15 (0)	-		
Eastern ghat high land	Warm and humid	Koraput	0/8 (0)	6/11 (54.54)	2/18 (11.11)	10/21 (47.62)
		Nowarangpur	2/10 (20)	4/10 (40)		
South Eastern ghat		Malkangiri	2/9 (22.22)	3/8 (37.5)	2/9 (22.22)	3/8 (37.5)
χ^2^ p value					11.855 0.22160	33.515 0.00001

## Discussion

Sheep pox and goat pox are economically important diseases affecting small ruminants of the Indian subcontinent, Middle East, Central, and Northern Africa with adverse effects to the mutton and wool producing countries [7]. In India, the nomadic habit of sheep and goats contributes to spreading of the infection into new areas. Movement of infected animals acts as the main source of spreading the infections [5]. Transmission also occurs mechanically by the propagation of insects such as *Stomoxys calcitrans* and the tsetse fly through a wider geographical area [14].

Several reports of seroprevalence of *Capripoxvirus* infections in India have previously been published, including one stating a 68.3% seropositivity in sheep from five northern states of India [13]. Through active (June 2007-May 2009) and passive surveillance (June 1997-May 2009), an incidence rate of 26.67%, 6.66%, 20.00%, and 46.67% for sheep pox was observed in North West agro-climatic zone of Tamil Nadu during summer, South West monsoon, North East monsoon, and winter seasons, respectively [15]. Similarly, the varied prevalence of goat pox among different agro-climatic zones has also been reported elsewhere [16].

Seroprevalence variation among sheep and goats according to agro-climatic zones as recorded in our study may be attributed to varying temperatures, humidity and rainfall, since the incidence of diseases may be influenced by climatic changes [17]. The stress resulting from climatic variations may act as a predisposing factor, as it reduces immunity to a certain extent, making the animals vulnerable to infections. The density of fly population and suitability for breeding with respect to different agro-climatic zones may also be one of the causes of variation. More numbers of capripox outbreaks are encountered during rainy season as compared to other time periods of the year [18].

Variations observed for different districts may be attributed to reasons similar to those listed above for agro-climatic zones. The results and significance of variations in both cases may change on increased sampling. The prevailing SPPV and GTPV infections in India resulted in restrictions imposed on international trade and exportation of small ruminants and their products, which in turn cause huge economic losses every year.

As no vaccination program is being carried out for these diseases in Odisha, the present study confirms the seroprevalence of *Capripoxvirus* infections among small ruminants due to disease outbreaks. Effective control programs demand socioeconomic and political stability, adequate infrastructure, functional veterinary services, and logistical support [9]. In India, quarantine and isolation are quite difficult due to the nomadic behavior of these animals, while stamping out and safe disposal of affected animals is also not possible. Restriction of animal movements in endemic areas with high seroprevalence, coupled with ring vaccination can significantly contribute to disease control. The vaccination strategies are considered to be the most economical and sustainable means of disease control in India [19]. Along with routine vaccination, active surveillance should be adopted at regular intervals to determine the spatial and temporal distribution of *Capripoxvirus* infections.

## Conclusion

An overall seroprevalence of 8.88% sheep pox and 31.47% goat pox were recorded in the study areas with significant variations among goats in different agro-climatic zones. Since vaccination is not practiced, the results provided serological evidence of *Capripoxvirus* infections in sheep and goat population of the state. Further, prevention strategies must be implemented to control the infection in the small ruminant population.

## Authors’ Contributions

SB, NS, and AH designed the study. AH collected serum samples across the state. MR helped in communication with Pox laboratory, IVRI, Mukteswar, and improvement of the manuscript through critical reading. SA processed and performed ELISA on the samples under the supervision of GV, AK, and MAR, who analyzed and interpreted the results. The whole work could be carried out with due permission from ABP. AH wrote the manuscript under the guidance of all authors. Finally, all authors interpreted, critically revised the manuscript and approved its final version.
